# Phenotypic Remodeling of γδ T Cells in Non-Eosinophilic Chronic Rhinosinusitis with Nasal Polyposis

**DOI:** 10.3390/medicina61122143

**Published:** 2025-11-30

**Authors:** Vjeran Bogović, Mario Štefanić, Stjepan Grga Milanković, Željko Zubčić, Hrvoje Mihalj, Stana Tokić, Martina Mihalj

**Affiliations:** 1 Department of Otorhinolaryngology and Head and Neck Surgery, Clinical Hospital Centre Osijek, J. Huttlera 4, HR-31000 Osijek, Croatia; bogovic.vjeran@kbco.hr (V.B.);; 2 Department of Otorhinolaryngology and Maxillofacial Surgery, Faculty of Medicine, Josip Juraj Strossmayer University of Osijek, HR-31000 Osijek, Croatia; 3 Department of Nuclear Medicine and Oncology, Faculty of Medicine, Josip Juraj Strossmayer University of Osijek, HR-31000 Osijek, Croatia; mstefanic@mefos.hr; 4 Department of Laboratory Medicine and Pharmacy, Faculty of Medicine, University of Osijek, J. Huttlera 4, HR-31000 Osijek, Croatia; 5 Department of Dermatology and Venereology, University Hospital Osijek, J. Huttlera 4, HR-31000 Osijek, Croatia; 6 Department of Physiology and Immunology, Faculty of Medicine, University of Osijek, J. Huttlera 4, HR-31000 Osijek, Croatia

**Keywords:** CRSwNP, flow cytometry, Th1, γδ T cells

## Abstract

*Background and Objectives*: Emerging evidence indicates that γδ T cells contribute to mucosal inflammation, yet their composition and functional characteristics in the nasal mucosa and nasal polyps remain insufficiently defined. This study aimed to characterize γδ T cell subsets in patients with non-eosinophilic chronic rhinosinusitis with nasal polyps (non-ECRSwNP) and to assess their associations with clinical features. *Materials and Methods*: Using flow cytometry, we analyzed the frequencies and phenotypes of γδ T cell subsets in middle nasal turbinate (MNT) tissue and nasal polyps from patients with non-eosinophilic chronic rhinosinusitis with nasal polyps (non-ECRSwNP), and compared them with nasal mucosa from healthy controls. Correlations with age, sex, disease severity, and allergy were also examined. *Results*: Distinct alterations in γδ T cell composition were observed in non-ECRSwNP, marked by a predominance of Vδ1^+^ and double-negative (DN) subsets. In nasal polyps, these shifts were influenced by age and sex, with a decline in Vδ1^+^ and a rise in Vδ2^+^ cells in older individuals showing decreased Vδ1^+^ and increased Vδ2^+^ cell frequencies, and males exhibiting consistently higher Vδ1^+^ levels. Higher disease severity (Lund–Mackay score > 12) was associated with an increased proportion of DN cells relative to Vδ1^+^ cells. *Conclusions*: There were observed alterations in γδ T cell subsets. These results suggest their potential role for certain γδ T cell subsets in non-ECRSwNP pathogenesis, warranting further studies on their function and involvement in the pathogenesis of non-ECRSwNP. These findings highlight the need for further studies to define the functional roles of these subsets across different stages of the disease.

## 1. Introduction

Chronic rhinosinusitis (CRS) is a common inflammatory disorder of the upper airways [1,2,3,4], defined by the presence of at least two symptoms such as nasal obstruction or discharge, in conjunction with facial pain or pressure and/or olfactory dysfunction lasting more than 12 weeks [4]. CRS is further classified into forms with nasal polyps (CRSwNP) or without nasal polyps (CRSsNP). Although initial management of CRSwNP is initially managed with medical therapy, many cases ultimately require surgical intervention due to a high recurrence rate. Within the CRSwNP subset, further classification reflects differences in immune cell infiltrate and associated effector mechanisms. Asian populations more frequently show neutrophilic, type 1-dominant immune response, while Western populations typically exhibit eosinophilic, type 2-driven disease (ECRSwNP) [5]. Despite progress in understanding CRSwNP immunopathology, the precise etiology and pathophysiological mechanisms driving polyp formation remain incompletely defined [6], reflecting complex contributions from genetics, epithelial barrier dysfunction, and microbial exposure and environmental factors [7,8].

Gamma delta (γδ) T cells are unconventional T lymphocytes enriched at mucosal and epithelial surfaces, where they participate in barrier defense and early immune surveillance [9,10,11,12]. Unlike αβ T cells, γδ T cells recognize non-peptide antigens without major histocompatibility complex (MHC) restriction and can rapidly produce cytokines that shape downstream immune responses [10,13]. In humans, γδ T cells are commonly classified by δ-chain usage into Vδ1 and Vδ2 subsets, which differ in tissue distribution, antigen responsiveness, and effector programs. Vδ1 and Vδ2 represent the dominant γδ T cell populations, whereas Vδ1^−^Vδ2^−^ variants expressing Vδ3–Vδ8 chains are rare and largely tissue-restricted. The circulating compartment is dominated by innate-like Vγ9Vδ2 cells responsive to phosphoantigens in a butyrophilin-dependent manner, whereas Vδ2^−^ populations, especially the more adaptive, tissue-enriched Vδ1^+^ subset, display more diverse TCR repertoires and expand in response to viral stimuli [12].

γδ T cells are enriched in the nasal mucosa and further expanded in both allergic and chronic rhinosinusitis. In allergic rhinitis, γδ T cells comprise approximately 25–30% of CD3+ T cells, with selective epithelial expansion in perennial allergic rhinitis (PAR), seasonal allergic rhinitis (SAR), and chronic infective rhinitis (CIR) [14]. Elevated γδ T cell levels have also been reported in the nasal mucosa of CRSwNP patients, including both eosinophilic (ECRSwNP) and non-eosinophilic (non-ECRSwNP) forms. While ECRSwNP features Th2-driven eosinophilic inflammation, non-ECRSwNP is characterized by neutrophilic, Th1/Th17-skewed responses [15,16,17]. Given their capacity to produce IL-17A and the distinct tissue tropism of δ-chain subsets, γδ T cells may contribute to neutrophilic inflammation [18] and fibrotic remodeling [19,20,21], but their distribution, phenotype, and role in neutrophilic non-ECRSwNP remain poorly addressed.

To address this, we analyzed Vδ1 and Vδ2 TCR expression in mononuclear cells from nasal polyps and mucosa, revealing subset-specific γδ T cell remodeling in non-ECRSwNP patients.

## 2. Materials and Methods

### 2.1. Study Design and Patient Selection

This study included 31 patients divided into two groups: the non-ECRSwNP group (*n* = 21) consisted of individuals undergoing functional endoscopic sinus surgery (FESS) at the University Hospital Centre Osijek ENT Clinic, from whom nasal polyp and middle nasal turbinate (MNT) biopsies were obtained. All the patients met diagnostic criteria for non-ECRSwNP per EPOS guidelines [22]. The control group (*n* = 10) comprised patients undergoing nasal surgery for unrelated indications (e.g., septoplasty or turbinate reduction) without CRSwNP; mucosal samples were collected from the middle turbinate. The exclusion criteria included known allergy; asthma; COPD; malignancy; salicylate sensitivity; or recent use (within one month) of corticosteroids, antibiotics, or antihistamines. Patient enrolment and the collection of patient-derived samples began in September 2021.

All the participants underwent nasal endoscopy (Malm classification), and non-ECRSwNP patients additionally received CT imaging of the paranasal sinuses. Blood samples were collected for total serum IgE, and nasal swabs were analyzed for eosinophils. Quality of life was assessed using standardized questionnaires (SNOT-20, SNOT-22, Nose Score, and Japanese Test). Demographic and clinical data, including comorbidities, age, sex, drug allergies, and smoking status, were recorded.

The study was approved by the Ethics Committee of the University Hospital Osijek (R2-7990/2021) and the Ethics Committee of the Faculty of Medicine, J. J. Strossmayer University of Osijek (15-61-47-23-10). The study complied with the standards set by the latest version of the Declaration of Helsinki. All the participants signed informed consent.

### 2.2. Histopathological Analysis

Non-ECRSwNP was defined as <10% eosinophils in the total leukocyte infiltrate of nasal polyp tissue [23]. Biopsies were fixed in 5% formalin, paraffin-embedded, and stained using hematoxylin–eosin or Gomori’s method. Eosinophil counts were performed in five eosinophil-rich fields at 400× magnification across five randomly selected sections. Histology was conducted at the Department of Pathology and Forensic Medicine, University Hospital Center Osijek.

### 2.3. Isolation and Immunophenotyping of γδ T Cells

The mucosal samples were stored in DMEM supplemented with 10% FBS, HEPES, antibiotics, and other standard additives, and then macerated, incubated (37 °C, 30 min, 50 rpm), and homogenized using a gentle MACS Dissociator (Miltenyi Biotec, Bergisch Gladbach, Germany). Suspensions were filtered (70 µm), and mononuclear cells were isolated via Lymphoprep density gradient centrifugation. Cell counts were performed using a Bürker–Türk chamber. Aliquots (1 × 10^6^ cells/mL) were frozen in FBS/DMSO (−80 °C) for subsequent analysis.

### 2.4. Flow Cytometry Staining and Analysis

Standard protocols available at https://www.thermofisher.com/tr/en/home/references/protocols/cell-and-tissue-analysis/flow-cytometry-protocol.html (accessed on 6 September 2021) were used for sample preparation and surface antigen staining for flow cytometry. Briefly, viability staining was performed using LIVE/DEAD™ Near-IR kits (Thermo Fisher, Carlsbad, CA, USA). Fc receptor blocking was achieved with Human TruStain FcX™ (BioLegend, San Diego, CA, USA). The cells were labeled with fluorochrome-conjugated antibodies: CD3ε-FITC (clone UCHT1), TCRγδ-PE-Cy7 (BioLegend), TCRVδ1-APC (eBiosciences, San Diego, CA, USA), and TCRVδ2-PerCP/Cy5.5 (BioLegend). PBS with 0.5–1% BSA and 0.1% NaN_3_ (pH ~7.4) was used throughout the experiment. The controls included single-color, FMO, unstained, and negative samples. Compensations were calculated using BD™ CompBeads (BD Biosciences, Milpitas, CA, USA).

Flow cytometric acquisition was performed on a BD FACSLyric™ system equipped with 488, 633, and 403 nm lasers. Data were analyzed using the FlowLogic software (v11.0, Inivai Technologies, Mentone, Australia).

### 2.5. Statistical Methods

#### 2.5.1. Statistical Data Analysis

Categorical data are presented as ratios, absolute, and relative frequencies. The numerical data were summarized by the median and the interquartile range. For two independent groups, the *t*-test was used for normally distributed data (Anderson–Darling test) and homogeneous variances (Levene’s test); otherwise, the Mann–Whitney test was used. The KruskalWallis test was applied to three or more groups (subgroup analysis), followed by Conover’s pairwise post hoc comparisons. Fisher’s exact test was applied to contingency tables. A two-tailed test with *p* < 0.05 was considered significant. The ggtern package was used to create the ternary plots (v3.5.0).

#### 2.5.2. Data Reduction

The patients were classified as having mild (≤12, low, *n* = 10) or severe (>12, high, *n* = 9) disease based on the median Lund–Mackay CT score. Log transformation was used to stabilize the variance and renormalize distributions when necessary (SNOT, IgE, and CRP levels). The presence of eosinophils in nasal smears was coded as a binary, Bernoulli variable (0/1, negative/positive). No missing data were encountered for any studied variable.

#### 2.5.3. Regression Modeling

Compositional data (γδ cell-type proportions) were jointly analyzed by running a Bayesian Dirichlet-multinomial regression model, which tests for differences in cell subsets against a set of continuous and categorical predictors. This choice was motivated by the power and flexibility of Bayesian modeling with modest samples, especially when case–control effects occur concurrently with other nuisance variables, resulting in complex and interconnected effects on cell dynamics. Unless otherwise stated, age and sex (F/M) were included as explanatory variables (fixed effects). If values of 0 and 1 were present in the data, a trafo argument from the R package DirichletReg (v0.7-1) was used to transform the proportion data [24]. Markov chain Monte Carlo sampling was used with a uniform number of iterations and a thinning scheme across all models (seed = 1234). All the models were run with four walkers, each taking 5000 steps, with a warm-up (burn-in) period of 2500 iterations (brms library, v2.20.4) [25,26]. Weakly informative prior distributions were employed on intercepts [student_t(3, 0, 2.5)], gradients [N(0, 10)], and phi [Γ(0.01, 0.01)] to let the data speak for themselves. We checked that varying these parameters does not have a significant impact on the results. Posterior convergence was assessed by Ȓ statistics (Ȓ = 1.00), effective sample size measures, and a visual inspection of trace plots. All the chains were deemed to have converged, and no divergent transitions were observed. We assessed the importance of the predictors using 95% credible intervals that were either strictly positive or strictly negative. The interpretation of Bayes factors (β), which quantify the statistical support for one model against another (typically the null-model, or the include/omit scenario), followed Kas and Raftery [27]: 1 < β ≤ 3—weak, 3 < β ≤ 20—positive, 20 < β ≤ 150—strong evidence (effectsize). We fitted a full model with all the samples and models based on subsets of the available samples (cases only, healthy only). Conditional effects were extracted and plotted as posterior draws of the expected value of the posterior predictive distribution. When creating conditional effects, the mean was used for continuous variables and the reference category was used for factors (sex: F; Site: controls; eosinophil: neg).

In order to test and control for bias due to age and sex, we also performed beta/binomial generalized linear (fixed/mixed) models using the glmmTBM R package (v1.1.10). This also provided a frequentist view of the Bayesian inference from the exploratory step [28]. A random intercept, nested in healthy/affected status, was included where necessary. An interaction term was fitted when possible to assess the homogeneity of effect at different ages in males and females. A detailed specification of each model is available in Supplementary (source) data. Model selection (comparison) was based on minimizing second-order Akaike Information Criteria AICc [29,30,31]. Near-equivalence of the competing models was indicated by ΔAICc < 2. The probability that a candidate model is the best in a set of competing models was quantified using Akaike weights (AICcmodavg, v2.3-4 package). The ggeffects (v2.3.1) package [32] was used to compute the marginal means and the adjusted predicted, covariate-adjusted values of the response. This allowed us to isolate and visualize the impact of each predictor, greatly simplifying the interpretation of results.

Data processing, analysis, and visualization were performed in R version 4.3.1. The following packages were also used: bayesplot v1.10.0, bayestestR v0.15.2, see v0.11.0, insight v1.4.2, tidyverse v2.0.0, targets v1.8.0, tidybayes v3.0.6, scales v1.3.0, patchwork v1.3.0, ggtext v0.1.2, ggh4x v0.2.8.9000, kableExtra v1.4.0, broom.mixed v0.2.9.5, corrplot v0.92xtra v1.4.0, broom.mixed v0.2.9.5, corrplot v0.92, data.table v1.15.4, table1 v1.4.3, ggpubr v0.6.0, modelsummary v2.2.0, corrplot v0.92, DescTools v0.99.52, effectsize v1.0.0, extraDistr v1.10.0, ggh4x v0.2.8.9000, ggpubr v0.6.0, ggtext v0.1.2, Hmisc v5.1-1, insight v1.4.2, kableExtra v1.4.0, marginaleffects v0.17.0, modelsummary v2.2.0, patchwork v1.3.0, rstan v2.32.3, rstanarm v2.26.1, scales v1.3.0, see v0.11.0, sjPlot v2.8.15, table1 v1.4.3, targets v1.8.0, tidybayes v3.0.6, tidyverse v2.0.0, and TMB v1.9.17. The codes supporting the results and figures are available upon reasonable request to the corresponding author.

## 3. Results

### 3.1. Subject Characteristics

A flow cytometric analysis was performed on tissue samples from 19 patients with non-ECRSwNP (min. age 17 yr, max. 76 yr) and 10 control subjects (min. age 20 yr, max. 51 yr). A significant difference in gender distribution was observed between the groups: the control group consisted predominantly of females, whereas the patient group consisted primarily of males (Table 1). Additionally, the control subjects were younger than the non-ECRSwNP patients. The non-ECRSwNP patients reported a significantly lower quality of life than healthy controls, as reflected by the results of the SNOT-20 and Nose Score questionnaires (Table 1). Among them, the patients with higher LM CT scores and women reported a greater symptom burden (Supplementary Table S1). There were no significant differences between the two groups regarding the serum IgE levels, C-reactive protein (CRP, 0.4–10.1 mg/L, min.–max.), or the prevalence of allergic hypersensitivity (Table 1).

### 3.2. γδ T Cell Distribution in Nasal Mucosa

A flow cytometric analysis was successfully completed on 35 tissue samples from affected individuals, comprising 19 nasal polyps and 16 middle nasal turbinate (MNT) specimens (in total, 14 paired samples) (Table 2).

**Figure 1:**
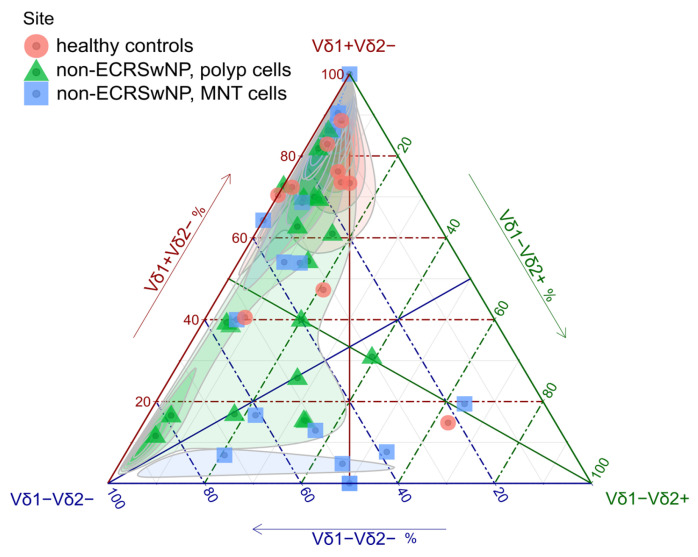
Ternary plot γδ T cell composition according to Vδ chain usage (flow cytometry; nasal mucosa). The colors/shapes correspond to group membership (vertical label). Each symbol represents one tissue sample. The plot is contoured according to the density of points across the triangle. The three solid grid lines bisect the triangle. Overall, the proportion of aggregate γδ cells in the nasal T cell population was similar to the proportion of γδ T cells found in the bloodstream [33]. However, their composition differed greatly: the nasal mucosa was primarily populated by Vδ1^+^Vδ2^−^ γδ T cells and Vδ1^−^Vδ2^−^ subsets (Table 2, Figure 1), in stark contrast to adult blood-borne γδ T cells, which typically carry the Vδ2 chain [33,34].

The ternary plots revealed the clustering of the control samples and their separation from polypoid mucosa, suggesting significant compositional differences along the Vδ1 and double-negative (Vδ1^−^Vδ2^−^) axes (Figure 1, Table 2). The distribution of the MNT samples was quite different and nearly bimodal: one group of cases (comprising half of all the samples) clustered with the controls; the remaining half displayed extensive, blood-like Vδ2 chain usage reminiscent of contaminating blood cells. This, combined with the consistently lower cell yield (and most likely, lower depth of surgical sampling), prompted us to exclude the MNT specimens from further consideration (22,150–165,978 vs. 8637–53,425 cells per sample, Mann–Whitney *p* = 0.046).

The total fraction of T cells and γδ T cells in the nasal mucosa was stable across age groups, sexes, and all biochemical covariates examined (Table 2 and Supplementary Table S2).

**Figure 2:**
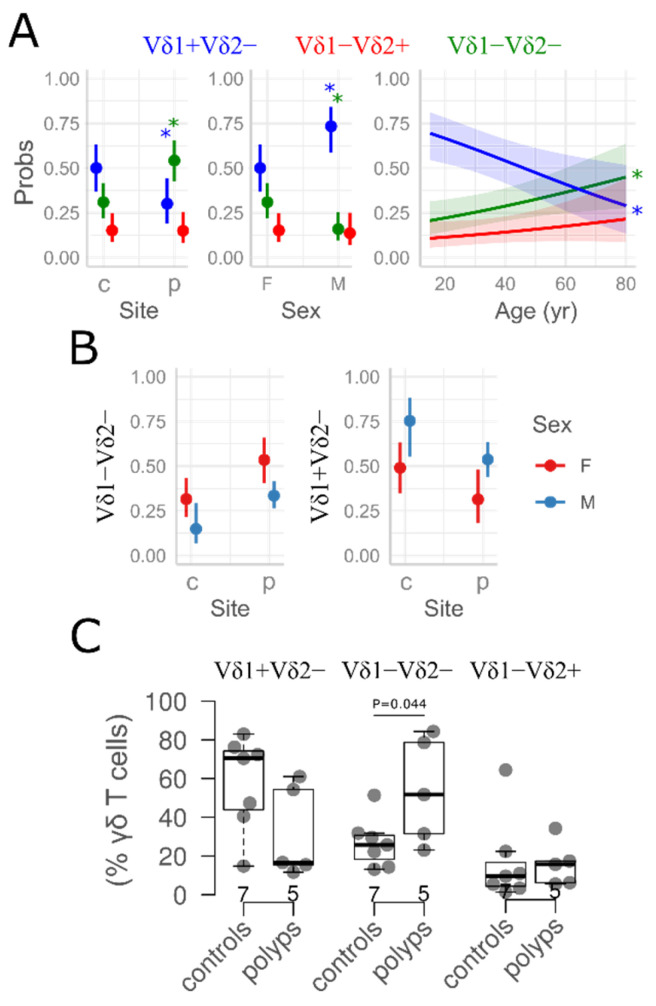
Marginal means (95% confidence interval): (**A**) Predictors of γδ T cell composition (generalized linear model, beta regression, fixed effects, and age + sex + case–control status). Each panel shows the independent, covariate-adjusted effect of the respective predictor. The Y-axis (Probs) shows the proportions of the γδ T cell pool occupied by each cell subset. For clarity, only significant effects are indicated. (**B**) The predicted proportions of Vδ1^−^Vδ2^−^ and Vδ1^+^Vδ2^−^ γδ T cells. The case–control differences were split by sex (Site × sex interaction term) and. Site denotes case (*N* = 19) − control (*N* = 10) status, polyps (p) vs. healthy mucosa (c). An asterisk denotes a significant difference (or effect), *p* < 0.05; color corresponds to cell type (**A**) or sex (**B**). For each cell type, the pairwise contrasts were based on female sex (M vs. F) and healthy donors as reference groups (p vs. c). Shaded areas correspond to 95% confidence intervals. F—females; M—males. For details on regression models and their numerical solutions, see Supplementary Tables S3 and S4. (**C**) Case–control comparisons (*t*-test) for each γδ T cell subset, raw data, female participants. Boxplots are defined by medians and their respective interquartile ranges (IQRs). Vertical lines extend to ±1.5 IQR. Yr years.

**Figure 3:**
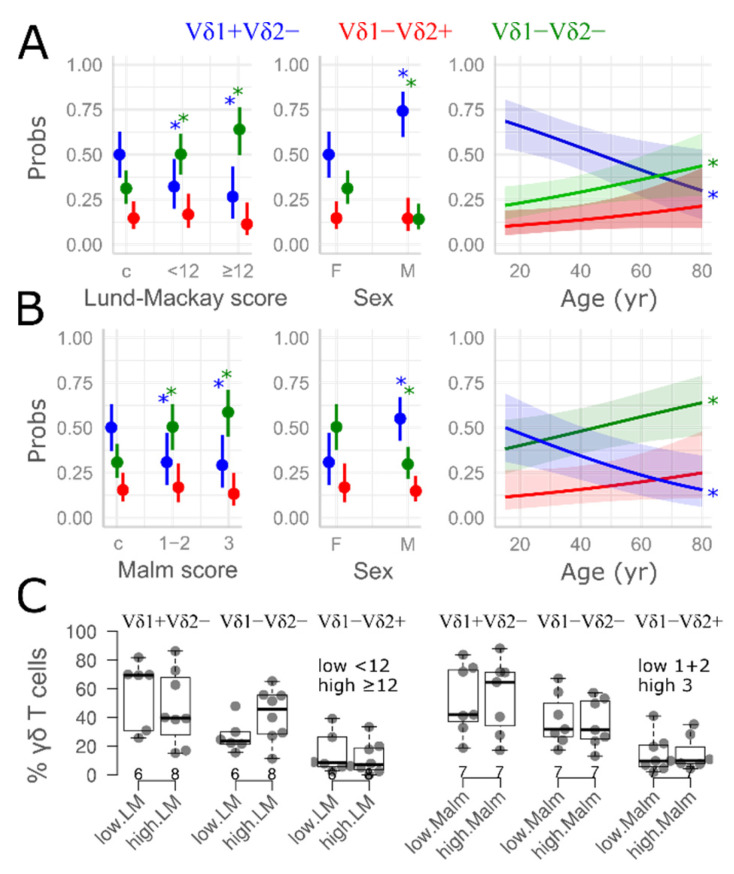
Marginal means (95% confidence interval), predictors of γδ T cell composition (generalized linear model, beta regression, and fixed effects). Each row corresponds to one age- and sex-adjusted regression model built around Lund–Mackay (LM) score (**A**), and Malm grade (**B**). The Y-axis (Probs) shows the predicted proportions of the γδ T cell pool occupied by each cell subset. An asterisk denotes a significant difference (or effect, *p* < 0.05); the pairwise contrasts are based on the following reference groups: female sex and healthy donors (c, controls). For clarity, only significant effects are indicated. Color corresponds to cell type (horizontal label EoI eosinophil index, nasal smears). Shaded areas correspond to 95% confidence intervals. F—females; M—males; pos—positive. For details on regression models and their numerical solutions, see Supplementary Tables S6–S8. (**C**) Cases (male participants), illustrating no effect of disease severity on γδ T cell composition in nasal polyps (raw data, Mann–Whitney *p* > 0.05, high vs. low comparisons). Boxplots are defined by medians and their respective interquartile ranges (IQRs). Vertical lines extend to ±1.5 IQR.

Similarly, there was no difference in the total number of T and γδ T cells between the healthy and affected, polypoid mucosa (Table 2 and Supplementary Table S2). Nevertheless, a robust and independent change in γδ cell-type composition was identified using different modeling approaches, all of which produced consistent results (Figure 2 and Supplementary Figure S1). Overall, a residual, disease-related, reciprocal shift in Vδ1^−^Vδ2^−^ and Vδ1^+^Vδ2^−^ subpopulations was found after adjusting for age and sex: Vδ1^+^Vδ2^−^ cells, which dominated the unaffected tissue, were partially replaced by Vδ1^−^Vδ2^−^ cell-types in polyps (Figure 2, Supplementary Figure S1, Supplementary Table S3). Consequently, the wide frequency gap between the Vδ1^+^Vδ2^−^ and subdominant Vδ1^−^Vδ2^−^ subsets diminished in the non-ECRSwNP mucosa [Vδ1^+^Vδ2^−^/Vδ1^−^Vδ2^−^ ratio: 3.87 (1.31–5.7) vs. 1.04 (0.3–2.83), healthy mucosa (*n* = 10) vs. polyps (*n* = 19), *p* = 0.042, Mann–Whitney test]. As a result, the Vδ1^+^Vδ2^−^ and Vδ1^−^Vδ2^−^ cells were more evenly distributed in nasal polyps. The exact details varied by sex and age: for instance, the mucosal samples obtained from men contained consistently more Vδ1^+^Vδ2^−^ cells and fewer Vδ1^−^Vδ2^−^ cells than those obtained from women under both conditions (Figure 2B, Supplementary Table S4). Consequently, men maintained a weak Vδ1^+^Vδ2^−^:Vδ1^−^Vδ2^−^ hierarchy in the polypoid (non-ECRSwNP) mucosa. Among the affected women, equilibration or reversal was common (Figure 2, Supplementary Tables S4 and S5). To a lesser extent, older age was also independently associated with the gradual replacement of the Vδ1^+^Vδ2^−^ subset by Vδ1^−^Vδ2^−^ cell-types (Figure 2 and Figure 3, and Supplementary Figure S1; Supplementary Table S3). Regardless of sex or age, however, the qualitative feature distinguishing the affected from the healthy mucosa (i.e., the exchange of Vδ1^+^Vδ2^−^ for Vδ1^−^Vδ2^−^ cells) was robust (β = 76.1, age + sex + CRS vs. age + sex; the corresponding ΔAICc are available in Supplementary Table S3). This confirmed that age and sex alone were insufficient to explain the observed compositional differences (β = 0.15, age + sex vs. null; Supplementary Table S3). The remaining proportion of Vδ1^−^Vδ2^+^ cells, on the other hand, was largely unaffected by age, sex, or non-ECRSwNP (Figure 2 and Figure 3, Supplementary Figure S1; Supplementary Table S3).

The severity of the disease, as determined by endoscopic (Supplementary Table S6) and radiologic scores (Supplementary Table S7), did not affect the results: quantitatively similar dynamics (decreased Vδ1^+^Vδ2^−^ and increased Vδ1^−^Vδ2^−^ fractions) were observed in both limited disease (Malm grades 1–2, low LM CT scores) and extensive disease (Malm grade 3, high LM CT scores) (Figure 3 and Supplementary Figure S1, Supplementary Table S5). Similarly, no significant associations were observed regarding subjective, patient-reported burden of symptoms or quality of life.

The resulting local Vδ1^−^Vδ2^−^ γδ profile—in turn—influenced the prevalence of eosinophil shedding at the sinonasal barrier (β = 44): the higher the Vδ1^−^Vδ2^−^ content, the more likely it was that eosinophils would emerge at mucosal surface (Supplementary Table S8) and shed into the nasal cavity, underscoring the functional relevance of the γδ niche (Supplementary Table S5, Supplementary Figure S2). IgE, age, and sex did not affect this relation (β = 0.18, age + sex vs. null). In this context, the connection between the granulocyte compartment and resident innate-like T cells at mucosal sites was not new, but remains poorly characterized in CRSwNP [35,36].

Finally, an additional modifying covariate was potentially identified by focusing solely on control samples. A higher serum IgE was associated with more Vδ1^−^Vδ2^+^ and fewer Vδ1^+^Vδ2^−^ γδ T cells within an otherwise stable γδ T cell population of healthy mucosa (β± = 85.9 vs. null-model, Vδ1^+^Vδ2^−^ fraction, Supplementary Figure S3, Supplementary Table S9). A directionally consistent trend was also seen in affected tissue (Supplementary Figure S3); however, the non-ECRSwNP cohort provided no reliable information about the magnitude of the effect (note that the latter was deliberately and selectively enriched for the non-eosinophilic variant of the disease, strongly disfavoring local type 2 inflammation). In such settings, the steroid-naive ECRSwNP cohort would provide a more appropriate testing ground for replication efforts.

## 4. Discussion

The unique functions of γδ T cells remain an active area of ongoing investigation, particularly given their preferential localization to peripheral tissues rather than lymphoid organs—an anatomical bias that may hold key immunological insights. This distinctive distribution is established during thymic development through sequential “waves” of γδ T cell maturation, which facilitate the efficient seeding of peripheral tissues by long-lived γδ T cells poised for rapid response [37,38,39]. These tissue-resident, γδ T cells function as an early line of defense against infectious pathogens and mediate innate-like immune responses, including direct cytotoxicity against infected or stressed cells, recruitment of neutrophils, activation of phagocytes, and promotion of granuloma formation [40]. Furthermore, their role spans health and disease. The potential role of γδ T cells in tumor immunosurveillance has recently been highlighted in a clinical study demonstrating promising outcomes following the allogeneic transfer of expanded Vδ2 γδ T cells [35,36,37].

In our study of non-ECRSwNP patients, the nasal mucosa contained proportions of γδ T cell comparable to peripheral blood; however, the tissue γδT cell compartment was dominated by Vδ1^+^ (D1) and double-negative (DN; Vδ1^−^Vδ2^−^) subsets rather than the Vδ2^+^ populations that predominate in circulation. Relative to the healthy nasal mucosa, the nasal polyp tissue exhibited a reciprocal compositional shift, with declining D1 frequencies and expanding DN cells, while the overall γδ T cell and CD3+ T cell numbers remained stable. These compositional changes were further shaped by demographic and clinical variables: D1 frequencies decreased with age, accompanied by a reciprocal rise in D2, remained consistently higher in male patients, and were markedly altered in patients with high Lund–Mackay scores (>12), who exhibited an augmented expansion of DN γδ T cells relative to D1 cells.

This mucosal predominance of Vδ1^+^ over Vδ2^+^ cells is also consistent with the patterns observed in chronic infections and cancer [40,41], where sustained antigenic stimulation drives adaptive expansion of Vδ1^+^ populations and contraction of more innate-like Vγ9Vδ2^+^ cells, the latter being prone to activation-induced cell death under chronic stress conditions [42]. Supporting the pathogenic potential of specific γδ subsets in upper-airway disease, Li et al. reported that Vγ1^+^ T cells are enriched in ECRSwNP [15] and correlate with heightened eosinophil infiltration, increased symptom burden, and markers of tissue remodeling [16]. Experimental depletion of Vγ1^+^ cells in an animal CRS model reduced eosinophilic inflammation and suppressed T2-associated cytokines (IL-4, IL-5, and IL-13) and GATA3 expression [15], indicating that defined γδ subsets can potentiate T2-biased inflammation. Human single-cell data from Wang et al. [17] further indicate that γδ T cells in CRSwNP acquire a memory–effector and cytotoxic profile, with elevated IL7R, GZMB/GZMH/GZMK, NKG7, PRF1, CXCR6, ITGA1, and ITGB1, supporting enhanced tissue residency, cytotoxicity, and chemotactic potential within polyp mucosa. These findings suggest that activated γδ niches may contribute to epithelial stress responses and sustained mucosal inflammation in eosinophilic disease. However, most mechanistic insights into γδ T cell function arise from murine Vγ-lineage subsets, which do not directly correspond to human δ-chain-defined populations, leaving the precise human subsets driving these effects unresolved. Given this uncertainty and the distinct inflammatory profile of non-ECRSwNP, further studies are required to determine whether similar γδ-driven pathways operate in non-eosinophilic disease or represent endotype-specific mechanisms.

The results of the present study revealed additional effects of age and sex on the γδ T cell composition. We observed an age-associated decline in the D1 subset, accompanied by a reciprocal rise in D2 γδ T cells, and consistently higher D1 proportions in male patients across all analyses. These findings align with prior studies reporting dynamic age-related restructuring of the γδ T cell pool. Namely, the Vγ9^+^Vδ2^+^ γδ T cell subset emerges early in fetal development, and becomes dominant in mid-gestation, displaying functional capacity of phosphoantigen-induced IFN-γ production [42,43,44,45,46]. After birth, the γδ T cell repertoire shifts toward greater representation of Vδ1^+^ cells, becoming more prominent [44], while Vδ3+ cells remain a minor subset in cord blood [43]. In adults, Vδ2^+^ T cells predominate in peripheral blood but decline after the age of 30 in patterns influenced by factors such as sex and ethnicity [47,48,49,50], and appear more resistant to immunosenescence compared to other γδ and αβ T cell subsets. Conversely, Vδ2^−^ populations (including Vδ1^+^ and Vδ1^−^Vδ2^−^) often acquire memory/effector phenotypes under chronic immune stimulation, such as CMV infection [51]. However, the age-associated decline in Vδ1^+^ cells observed in our study revealed an age-associated decline in Vδ1^+^ cells within the nasal mucosa samples, accompanied by a reciprocal rise in Vδ2^+^ frequencies, which represents an inverse pattern to that typically described in peripheral blood. Nevertheless, our findings align with a recent work by Gray JI et al., who reported similar age-associated reductions in Vδ1^+^ T cells and opposing increases in Vδ2^+^ cells across the blood, gut, and lung mucosa [52]. Additionally, they observed an age-related accumulation of memory γδ T cells with tissue-resident phenotypes, likely reflecting antigenic exposures over childhood-driven functional evolution, and tissue-specific maturation trajectories [52]. Collectively, these observations support the concept that γδ T cell differentiation and distribution are compartment-specific. Considering that Vδ1^+^ T cells are enriched in peripheral tissues rather than circulation, further studies are warranted to clarify how aging influences their functional roles across different anatomical compartments and how these shifts influence chronic mucosal inflammation. Sex-associated differences provide additional context for our findings. Sanz M et al. demonstrated a significant sex-specific difference in the composition of peripheral γδ T cells, with a more rapid decline in Vδ2^+^ T cells observed in women over the age of 40 [53]. In contrast, our dataset revealed that male subjects consistently exhibited higher proportions of Vδ1^+^ T cells in nasal polyps. These tissue-specific patterns differ from male patients, highlighting the divergence between mucosal and blood compartments and emphasizing the necessity of incorporating sex as a biological variable in mucosal immunology studies.

Within this context, the functional role of the DN (Vδ1^−^Vδ2^−^) subset in non-ECRSwNP remains largely speculative. DN cells likely include minor populations such as Vδ3+ and other rare δ-chain subsets enriched in tissues [54,55,56,57] but insufficiently characterized due to low abundance and limited reagents targeting TRVD3-8 TCRs. Vδ3^+^ T cells have been shown to facilitate B cell maturation by inducing CD40, CD86, and HLA-DR expression and promoting IgM secretion [58], a mechanism particularly relevant in nasal polyp tissue, where B cells and plasma cells generate local immunoglobulin responses [17,22], including IgE that sensitizes resident mast cells [17,58]. In line with this immunological milieu, we observed that higher DN frequencies correlate with increased eosinophil shedding at the sinonasal surface, independent of IgE levels, age, or sex. This pattern raises the possibility that DN-enriched γδ niches could influence granulocyte recruitment via cytokine-driven interactions with B cells or innate effector populations, rather than by directly promoting IgE production. This model aligns with experimental evidence from animal studies demonstrating that γδ T cells can activate eosinophils under tissue-stress conditions [35,36], though the mechanism in human CRSwNP remains uncharacterized. Finally, the modest clinical efficacy of eosinophil-depleting therapies [59] relative to interventions targeting IgE or IL-4Rα signaling [60] further highlights the relevance of B cell-mediated pathways in polyp biology.

Finally, a small sample size remains a major issue. As a result, a number of simplifying assumptions were made, which precluded the use of more elaborate explanatory models. Consequently, the estimated compositional shifts are still subject to significant uncertainty, indicating the presence of unmodeled features in the data. Ultimately, selection effects must also be considered, given the well-established referral bias toward more severe disease presentations. Thus, no causal inferences should be drawn from these data.

## 5. Conclusions

This study demonstrates that the γδ T cell compartment in non-ECRSwNP nasal mucosa and polyps is dominated by Vδ1^+^ and double-negative (Vδ1^−^Vδ2^−^) subsets, with reciprocal shifts in these subsets relative to healthy tissue. Age- and sex-related differences further shape their composition. Extending prior studies that largely focused on peripheral blood, these findings provide novel insights into tissue-specific γδ T cell remodeling in chronic sinonasal inflammation. While the functional role of the double-negative subset remains speculative, its association with mucosal immune activity raises intriguing questions regarding potential interactions with B cells and granulocytes, warranting targeted validation in the human eosinophilic form of CRSwNP.

## Figures and Tables

**Table 1:** Demographic, biochemical, and clinical data by case–control status (non-eosinophilic chronic rhinosinusitis with nasal polyps, non-ECRSwNP).

	Controls	Polyps, Non-ECRSwNP	MannWhitney *p*
	(*n* = 10)	(*n* = 19)	
Age (yr)	42 [29, 48]	54 [35, 65]	0.108
SNOT22	11 [2, 20.25]	35 [23, 53]	0.224
SNOT20	29 [15, 40.25]	41 [27, 60]	0.0078
Japan	13 [5, 26]	22 [13, 46]	0.108
Nose.score	7 [5, 10]	16 [13, 17]	0.0069
Lund–Mackay score	-	11 [8.0, 16]	-
IgE (pg/mL)	62.50 [26, 114.3]	115 [37.5, 271]	0.347
CRP (mg/L)	1.3 [0.68, 1.83]	1.84 [1.39, 2.95]	0.148
Sex			0.046 *
F	7 (70.0%)	5 (26.3%)	
M	3 (30.0%)	14 (73.7%)	
LMI			
controls	10 (100%)	0 (0%)	-
high	0 (0%)	9 (47.4%)	
low	0 (0%)	10 (52.6%)	
Malm grade			-
controls	10	0	
1	0	3	
2	0	7	
3	0	9	
Inhalant allergens			0.236 *
neg	4 (40.0%)	13 (68.4%)	
pos	6 (60.0%)	6 (31.6%)	
Nutritive allergens			0.298 *
neg	10 (100%)	16 (84.2%)	
pos	0 (0%)	3 (15.8%)	
Eosinophils			0.431 *
neg	8 (80.0%)	12 (63.2%)	
pos	2 (20.0%)	7 (37%)	

* Fisher’s exact test. Lund-Mackay (LM) low ≤ 12; LMI high ≥ 12. IgE serum immunoglobulin E. CRP C-reactive protein. Numerical data are presented as median with interquartile range.

**Table 2:** Flow cytometry cell counts according to sampling site and case–control status (non-eosinophilic chronic rhinosinusitis with nasal polyps, non-ECRSwNP).

Cell Population	Controls	p, Non-ECRSwNP	MNT, Non-ECRSwNP	Mann–Whitney *p* (p vs. Controls)
	(*N* = 10)	(*N* = 19)	(*N* = 16)	
Ly (% Parent)	40 [30, 50]	40 [40, 60]	30 [20, 40]	0.63
[Min, Max]	[20, 70]	[10, 70]	[10, 70]	
Ly (% Total)	30 [20, 40]	30 [20, 40]	20 [20, 30]	0.945
[Min, Max]	[20, 50]	[5, 60]	[8, 40]	
T (%Parent)	40 [40, 50]	50 [30, 60]	30 [10, 50]	0.909
[Min, Max]	[6, 80]	[5, 80]	[6, 80]	
T (% Total)	10 [8, 20]	10 [7, 20]	5 [4, 8]	0.872
[Min, Max]	[3, 30]	[2, 30]	[1, 20]	
γδ (% T)	4 [3, 8]	4 [3, 7]	4 [3, 6]	0.872
[Min, Max]	[2, 10]	[2, 20]	[0.9, 20]	
γδ (% ly)	0.5 [0.2, 1]	0.5 [0.3, 0.7]	0.3 [0.08, 0.4]	0.89
[Min, Max]	[0.09, 3]	[0.1, 2]	[0.03, 2]	
Vδ1^−^Vδ2^+^ (%γδ)	9 [4, 10]	8 [5, 20]	20 [5, 50]	0.646
[Min, Max]	[0, 60]	[0, 40]	[0, 60]	
Vδ1^+^Vδ2^−^ (%γδ)	70 [50, 80]	40 [20, 70]	30 [7, 70]	0.037
[Min, Max]	[10, 90]	[10, 90]	[0, 100]	
Vδ1^−^Vδ2^−^ (%γδ)	20 [10, 30]	30 [20, 50]	40 [20, 50]	0.018
[Min, Max]	[7, 50]	[10, 80]	[0, 70]	
Vδ1^−^Vδ2^+^ (% T)	0.3 [0.2, 0.5]	0.4 [0.2, 1]	0.5 [0.3, 1]	0.748
[Min, Max]	[0, 2]	[0, 3]	[0, 4]	
Vδ1^+^Vδ2^−^ (% T)	2 [1, 6]	2 [0.8, 3]	1 [0.3, 3]	0.302
[Min, Max]	[0.5, 10]	[0.4, 10]	[0, 10]	
Vδ1^−^Vδ2^−^ (% T)	0.9 [0.6, 2]	2 [1, 3]	1 [0.9, 2]	0.162
[Min, Max]	[0.3, 2]	[0.4, 5]	[0, 6]	

p—polyp; MNT—middle nasal turbinate mucosa; Ly—lymphocyte. Numerical data are presented as median with interquartile range. *p* < 0.05 is considered significant.

## Data Availability

The raw data supporting the conclusions of this article will be made available by the authors on request due to privacy concerns.

## Supplementary Materials

The following supporting information can be downloaded at https://www.mdpi.com/article/10.3390/medicina61122143/s1.
