# Supplementary material for: Phenotypic Remodeling of γδ T Cells in Non-Eosinophilic Chronic Rhinosinusitis with Nasal Polyposis
Source: Medicina (Kaunas). 2025 Nov 30;61(12):2143. doi: 10.3390/medicina61122143 (PMC12734732; doi:10.3390/medicina61122143)
Supplement: Supplementary file 1 [file medicina-61-02143-s001.zip › Suplementary Tables_correction_28.11.2025.pdf]

Phenotypic Remodeling of γδ T Cells in Non-Eosinophilic Chronic Rhinosinusitis with Nasal Polyposis

Vjeran Bogović [1,2]†, Mario Štefanić [3]†, Stjepan Grga Milanković[1,2,] ,Željko Zubčić[1,2], Hrvoje Mihalj[1,2], Stana Tokić[4,*], Martina Mihalj[5,6,*]

Supplementary Tables

Supplementary Table S1. Predictors for patient-reported quality of life (SNOT20 questionnaire), generalized linear regression model (case-group only).

	log(1+SNOT 20)		
Predictors	Estimates	95% CI	p
(Intercept)	2.76	0.75 – 4.76	**0.007**
LM [log]	0.96	0.41 – 1.50	**0.001**
Sex [M]	-0.95	-1.75 – -0.16	**0,019**
Age	-0,01	-0.03 – 0.01	0,362
logIgE	-0,2	-0.76 – 0.36	0,475
CRP [log]	-0,14	-0.70 – 0.42	0,617
Observations:	19		
R^2 / R^2 adjusted	0.486 / 0.230		

	log(1+SNOT 20)		
Predictors	Estimates	95% CI	p
(Intercept)	2,11	0.64 – 3.59	**0,005**
LM [log]	1,01	0.47 – 1.55	**<0.001**
Sex [M]	-0,92	-1.67 – -0.17	**0,016**
Age	-0,01	-0.03 – 0.01	0,352
Observations:	19		
R^2/R^2 adjusted	0.462 / 0.308		

LM Lund-Mackay CT score
IgE serum immunoglobulin E level (pg/mL)
CRP serum C-reactive protein (mg/L)
Reference categories (Sex:F)
CI confidence interval
R^2 coefficient of determination
Estimates represent regression coefficients
Bold font denotes $p < 0.05$

Table S2a. Predictors of total γδ T cell fraction (% T cells)

Generalized linear mixed model, beta-regression, fixed effects

	γδ (% Parent)		
Predictors	Estimates	95% CI	p
(Intercept)	-2,43	-3.03 – -1.82	**<0.001**
Site [p]	0,1	-0.38 – 0.58	0,687
Age	-0,01	-0.02 – 0.00	0,112
Sex [M]	0,06	-0.43 – 0.56	0,808
Observations:	29		
R^2	0,103		

Generalized linear mixed model, beta-regression, case-control status (Site) is modelled as random intercept

	γδ (% Parent)		
Predictors	Estimates	95% CI	p
(Intercept)	-2,58	-3.35 – -1.81	**<0.001**
IgE [log]	-0,05	-0.21 – 0.11	0,539
**Random Effects**			
σ^2	0,32		
$\tau_{00\ Site}$	0		
N_{Site}	2		
Observations:	29		
Marginal R^2 / Conditional R^2	0.016 / NA		

Generalized linear mixed model, beta-regression, the effect of serum IgE was split by case-control status (interaction term)

	γδ (% Parent)		
Predictors	Estimates	95% CI	p
(Intercept)	-2,34	-3.63 – -1.04	**<0.001**
logIgE	-0,26	-0.95 – 0.43	0,459
Site [p]	-0,38	-2.01 – 1.25	0,649
logIgE × Site [p]	0,21	-0.62 – 1.05	0,615
Observations:	29		
R^2	0,027		

Reference categories (Site: controls, Sex:F)

p polyp

CI confidence interval

R2 coefficient of determination

Estimates represent regression coefficients

Bold font denotes $p < 0.05$

ICC intraclass correlation coefficient

IgE immunoglobulin E

Table S2b. Predictors of total T cell fraction (% ly)

Generalized linear mixed model, beta-regression, fixed effects

	T (% Parent)		
Predictors	Estimates	95% CI	p
(Intercept)	1,31	0.54 – 3.18	0,544
Site [p]	1,07	0.54 – 2.11	0,851
Age	0,98	0.96 – 1.00	0,096
Sex [M]	1,31	0.67 – 2.55	0,432
Observations: 29			
R^2 = 0,091			

Generalized linear mixed model, beta-regression, case-control status (Site) is modelled as random intercept

		T (% Parent)		
Predictors		Estimates	95% CI	p
(Intercept)		0,08	-0.93 – 1.09	0,873
logIgE		-0,18	-0.67 – 0.30	0,464
**Random Effects**				
σ^2	0,2			
τ_{00} Site	0			
N Site	2			
Observations: 29				
Marginal R^2 / Conditional R^2	0.058 / NA			

Predicted gdT cell frequencies by site and IgE level, log IgE: 1.38

```
Site | Predicted | 95% CI
-----------------------------
ctrl |      0.06 | 0.04, 0.10
p    |      0.06 | 0.04, 0.09
```

logIgE: 1.99

```
Site | Predicted | 95% CI
-----------------------------
ctrl |      0.05 | 0.04, 0.08
p    |      0.06 | 0.04, 0.07
```

logIgE: 2.6

```
Site | Predicted | 95% CI
-----------------------------
ctrl |      0.05 | 0.02, 0.09
p    |      0.06 | 0.04, 0.08
```

p polyp, ctrl controls

CI confidence interval

Table S3. γδ T cell composition by case-control status (Site), age and sex (generalized linear model, beta-regression output). Each cell subset was modelled separately.

	Vδ1−Vδ2−		
Predictors	Estimates	95% CI	p
(Intercept)	-1,61	-2.33 – -0.89	**<0.001**
Site [p]	0,97	0.40 – 1.54	**0,001**
Age	0,02	0.00 – 0.03	**0,02**
Sex [M]	-0,86	-1.40 – -0.33	**0,001**

N: 29
$R^2 = 0,469$

	Vδ1+Vδ2−		
Predictors	Estimates	95% CI	p
(Intercept)	1,22	0.37 – 2.07	**0,005**
Site [p]	-0,85	-1.51 – -0.18	**0,013**
Age	-0,03	-0.05 – -0.01	**0,006**
Sex [M]	1,01	0.34 – 1.69	**0,003**

N: 29
$R^2 = 0,417$

	Vδ1−Vδ2+		
Predictors	Estimates	95% CI	p
(Intercept)	-2,3	-3.25 – -1.35	**<0.001**
Site [p]	-0,01	-0.79 – 0.76	0,976
Age	0,01	-0.01 – 0.03	0,221
Sex [M]	-0,12	-0.81 – 0.57	0,73

N: 29
$R^2 = 0,075$

Reference categories (Site: controls, Sex:F)
p polyp
CI confidence interval
R^2 coefficient of determination
Estimates represent regression coefficients

Model comparison, the second-order Akaike information criterion (AICc, Vδ1−Vδ2−)

Model	df	AICc	ΔAICc	AICcWT
Intercept-only	2	13,88	8,86	0,01
Age+Sex	4	-15,68	7,06	0,03
**Age+Sex+CRS(Site)**	**5**	**-22,74**	**0**	**0,96**

Model comparison, the second-order Akaike Information Criterion (AICc, Vδ1+Vδ2−)

Model	df	AICc	ΔAICc	AICcWT
Intercept-only	2	-1,44	6,7	0,03
Age+Sex	4	-5,21	2,93	0,18
**Age+Sex+CRS(Site)**	**5**	**-8,14**	**0**	**0,79**

df degrees of freedom
The Akaike weights (AICcWT) represent the probability that the candidate model is the best among the set of competing models.
Bold font denotes best-fit model.

Table S4. γδ T cell composition, the effect of case-control status (Site) was split by sex (generalized linear model, beta-regression)

	Vδ1-Vδ2-		
Predictors	Estimates	95% CI	p
(Intercept)	-1.59	-2.33 – -0.85	**<0.001**
Site [p]	0.91	0.18 – 1.65	**0.015**
Sex [M]	-0.97	-1.97 – 0.03	0.058
Age	0.02	0.00 – 0.03	**0.019**
Site [p] × Sex [M]	0.14	-1.02 – 1.31	0.81

Observations: 29

R^2 = 0.472

	Vδ1+Vδ2-		
Predictors	Estimates	95% CI	p
(Intercept)	1.19	0.32 – 2.05	**0.007**
Site [p]	-0.74	-1.64 – 0.16	0.106
Sex [M]	1.16	0.07 – 2.24	**0.037**
Age	-0.03	-0.05 – -0.01	**0.006**
Site [p] × Sex [M]	-0.23	-1.55 – 1.10	0.739

Observations: 29

R^2 = 0.421

Estimates represent regression coefficients

CI confidence interval

R^2 coefficient of determination

Reference categories (Site: controls, Sex:F)

Predicted cell frequencies by site and sex

```
Sex: F                  DN
Site | Predicted |    95% CI
-----------------------------
ctrl |      0.32 | 0.22, 0.43
p    |      0.54 | 0.41, 0.66

Sex: M
Site | Predicted |    95% CI
-----------------------------
ctrl |      0.15 | 0.07, 0.29
p    |      0.34 | 0.26, 0.41
Adjusted for:
Age = 46.00
```

```
Sex: F                  D1
Site | Predicted |    95% CI
-----------------------------
ctrl |      0.49 | 0.35, 0.63
p    |      0.31 | 0.18, 0.48

Sex: M
Site | Predicted |    95% CI
-----------------------------
ctrl |      0.75 | 0.55, 0.88
p    |      0.54 | 0.44, 0.63
Adjusted for:
Age = 46.00
```

p polyp, ctrl controls, CI confidence interval

Table S5. The average marginal effect for different Bayesian Dirichlet models (fixed effects, γδ T cell composition according to Vδ chain usage)

Case-control status (Site)					
Group	Term	Contrast	Estimate	2.50%	97.50%
Vδ1+Vδ2-	Age	+1	-0.00606	-0.00949	-0.00221
Vδ1-Vδ2+	Age	+1	0.00191	-0.00052	0.00471
Vδ1-Vδ2-	Age	+1	0.00408	0.000538	0.00747
Vδ1+Vδ2-	Sex	M - F	0.20759	0.075176	0.32045
Vδ1-Vδ2+	Sex	M - F	-0.02077	-0.10856	0.06255
Vδ1-Vδ2-	Sex	M - F	-0.18571	-0.30279	-0.05743
Vδ1+Vδ2-	Site	p - c	-0.19229	-0.31545	-0.05544
Vδ1-Vδ2+	Site	p - c	0.00508	-0.09779	0.0947
Vδ1-Vδ2-	Site	p - c	0.18892	0.063761	0.30453

Disease severity (Lund-Mackay, LM CT score)					
Group	Term	Contrast	Estimate	2.50%	97.50%
Vδ1+Vδ2-	Age	+1	-0.00583	-9.34E-03	-0.00201
Vδ1-Vδ2+	Age	+1	0.00198	-3.16E-04	0.00452
Vδ1-Vδ2-	Age	+1	0.00375	7.51E-05	0.00731
Vδ1+Vδ2-	LMI	h - c	-0.23236	-3.82E-01	-0.06865
Vδ1+Vδ2-	LMI	l - c	-0.17322	-3.07E-01	-0.02804
Vδ1-Vδ2+	LMI	h - c	-0.03095	-1.34E-01	0.07137
Vδ1-Vδ2+	LMI	l - c	0.02909	-8.01E-02	0.13143
Vδ1-Vδ2-	LMI	h - c	0.26455	1.08E-01	0.40614
Vδ1-Vδ2-	LMI	l - c	0.14499	1.42E-02	0.27426
Vδ1+Vδ2-	Sex	M - F	0.21885	8.97E-02	0.334
Vδ1-Vδ2+	Sex	M - F	-0.00511	-9.48E-02	0.07557
Vδ1-Vδ2-	Sex	M - F	-0.21238	-3.27E-01	-0.08275

Disease severity (endoscopic Malm score)					
Group	Term	Contrast	Estimate	2.50%	97.50%
Vδ1+Vδ2-	Age	+1	-0.00605	-0.00939	-0.00216
Vδ1-Vδ2+	Age	+1	0.00217	-0.00033	0.00503
Vδ1-Vδ2-	Age	+1	0.0038	0.000161	0.00712
Vδ1+Vδ2-	MalmI	h - c	-0.21262	-0.35963	-0.04807
Vδ1+Vδ2-	MalmI	l - c	-0.17723	-0.31818	-0.02493
Vδ1-Vδ2+	MalmI	h - c	-0.01535	-0.12468	0.09164
Vδ1-Vδ2+	MalmI	l - c	0.01891	-0.09068	0.12853
Vδ1-Vδ2-	MalmI	h - c	0.22668	0.074103	0.3679
Vδ1-Vδ2-	MalmI	l - c	0.15822	0.018936	0.2919
Vδ1+Vδ2-	Sex	M - F	0.21463	0.085158	0.33012
Vδ1-Vδ2+	Sex	M - F	-0.02518	-0.11355	0.05967
Vδ1-Vδ2-	Sex	M - F	-0.18744	-0.30537	-0.06325

Columns: term, group, contrast, estimate, conf.low, conf.high

Type: response

conf.low, conf.high correspond to 95% credible intervals for the contrast between different categories

Red font indicates strictly positive or strictly negative 95 % credible intervals

h high, l low, c controls, p polyps, M male, F female

Malm low = grade 1+2, Malm high = grade 3

LM low <12, LM high >=12

Table S6. γδ T cell composition by endoscopic disease severity (Malm Index, MalmI), age and sex (generalized linear model, beta-regression). Patients were classified as severely affected (Malm 3) or mildly affected (Malm1-2).

	**Vδ1−Vδ2−**		
Predictors	Estimates	95% CI	p
(Intercept)	-1,56	-2.27 – -0.85	**<0.001**
MalmI [h]	1,16	0.52 – 1.80	**<0.001**
MalmI [l]	0,83	0.22 – 1.44	**0,008**
Age	0,02	0.00 – 0.03	**0,029**
Sex [M]	-0,88	-1.40 – -0.36	**0,001**
Observations: 29			
R^2 = 0.492			

	**Vδ1+Vδ2−**		
Predictors	Estimates	95% CI	p
(Intercept)	1,21	0.35 – 2.07	**0,006**
MalmI [h]	-0,89	-1.66 – -0.12	**0,024**
MalmI [l]	-0,81	-1.54 – -0.08	**0,029**
Age	-0,03	-0.05 – -0.01	**0,008**
Sex [M]	1,01	0.34 – 1.69	**0,003**
Observations: 29			
R^2 = 0.419			

	**Vδ1−Vδ2+**		
Predictors	Estimates	95% CI	p
(Intercept)	-2,36	-3.33 – -1.40	**<0.001**
MalmI [h]	-0,17	-1.07 – 0.73	0,711
MalmI [l]	0,11	-0.72 – 0.94	0,79
Age	0,01	-0.01 – 0.04	0,18
Sex [M]	-0,16	-0.84 – 0.53	0,657
Observations: 29			
R^2 = 0.085			

Reference categories (MalmI: controls, Sex:F)

CI confidence interval

R^2 coefficient of determination

Estimates represent regression coefficients

Table S7. γδ T cell composition by radiological disease severity (Lund-Mackay CT score, LMI), age and sex (generalized linear model, beta-regression). Patients were classified by median LM score as severely affected (high, h) or mildly affected (low, l).

	Vδ1–Vδ2–		
Predictors	Estimates	95% CI	p
(Intercept)	-1,52	-2.21 – -0.82	**<0.001**
LMI [h]	1,37	0.70 – 2.03	**<0.001**
LMI [l]	0,8	0.22 – 1.38	**0,007**
Age	0,02	0.00 – 0.03	**0,031**
Sex [M]	-1,02	-1.55 – -0.49	**<0.001**
Observations: 29			
$R^2 = 0.533$			

	Vδ1+Vδ2–		
Predictors	Estimates	95% CI	p
(Intercept)	1,16	0.30 – 2.02	**0,008**
LMI [h]	-1,02	-1.82 – -0.22	**0,013**
LMI [l]	-0,75	-1.46 – -0.04	**0,039**
Age	-0,03	-0.04 – -0.01	**0,01**
Sex [M]	1,06	0.38 – 1.73	**0,002**
Observations: 29			
$R^2 = 0.43$			

	Vδ1–Vδ2+		
Predictors	Estimates	95% CI	p
(Intercept)	-2,38	-3.32 – -1.45	**<0.001**
LMI [h]	-0,3	-1.21 – 0.62	0,525
LMI [l]	0,16	-0.63 – 0.94	0,696
Age	0,01	-0.01 – 0.03	0,167
Sex [M]	-0,01	-0.70 – 0.67	0,967
Observations: 29			
$R^2 = 0.13$			

Reference categories (LMI: controls, Sex:F)

CI confidence interval

R2 coefficient of determination

Estimates represent regression coefficients

Bold font denotes $p < 0.05$

Table S8. The relationship between the γδ T cell composition (Vδ1−Vδ2− subtype) and the presence/absence of eosinophils in nasal smear (EoI, pos/neg). Only significant findings are reported. Cases and controls were modelled together (note that Vδ1−Vδ2− frequency already contains an information on case-control status).

Generalized linear model, beta-regression, fixed effects

	Vδ1-Vδ2-		
Predictors	Estimate	95% CI	p
(Intercept)	-1.5	-1.84 – -1.16	**<0.00**
EoI [pos]	1.1	0.46 – 1.74	**0.001**
Observations	10		
R^2	0.476		

Reference categories (EoI:neg, Sex:F)

CI confidence interval

R^2 coefficient of determination

Estimates represent regression

Bold font denotes $p < 0.05$

Generalized linear mixed model, beta-regression, case-control status (Site) is modelled as random intercept

	Vδ1-Vδ2-		
Predictors	Esti	95% CI	p
(Intercept)	0.2	0.08 – 0.51	**0.001**
Age	1.02	1.01 – 1.04	**0.003**
Sex [M]	0.46	0.28 – 0.77	**0.003**
EoI [pos]	1.94	1.18 – 3.18	**0.008**
**Random Effects**			
σ^2	0.15		
$\tau_{00\ Site}$	0.12		
ICC	0.44		
N_{Site}	2		
N	29		
Marginal R^2 / Conditional R^2	0.519 / 0.730		

Reference categories (EoI:neg, Sex:F)

ICC intraclass correlation coefficient

The regression coefficients were exponentiated.

Binomial regression

	EoI		
Predictors	Log-	95% CI	p
(Intercept)	-1.32	-3.96 – 1.32	0.326
Vδ1-Vδ2-	8.91	0.75 – 17.0	**0.032**
Sex [M]	1.28	-1.07 – 3.62	0.285
Age	-0.08	-0.17 – 0.01	0.067
Observations	29		
R^2 Tjur	0.28		

Reference categories (EoI:neg, Sex:F)

R^2 Tjur coefficient of discrimination

Estimates represent regression coefficients

Table S9. γδ T cell composition in relation to serum immunoglobulin E (IgE) levels in controls

	Vδ1+Vδ2−		
Predictors	Estimates	95% CI	p
(Intercept)	15,63	4.08 – 59.96	**<0.001**
Sex [M]	2,07	0.90 – 4.77	0,086
Age	0,99	0.96 – 1.03	0,717
IgE [log]	0,61	0.48 – 0.77	**<0.001**
Observations: 10			
$R^2 = 0.769$			

	Vδ1−Vδ2+		
Predictors	Estimates	95% CI	p
(Intercept)	0	0.00 – 0.02	**<0.001**
Age	1,06	0.99 – 1.14	0,115
Sex [M]	0,66	0.18 – 2.41	0,531
logIgE	2,7	1.23 – 5.93	**0,013**
Observations: 10			
$R^2 = 0.503$			

	Vδ1−Vδ2−		
Predictors	Estimates	95% CI	p
(Intercept)	0,42	0.12 – 1.40	0,156
Age	0,98	0.95 – 1.02	0,319
Sex [M]	0,59	0.23 – 1.50	0,267
logIgE	1,29	0.78 – 2.14	0,317
Observations:			
$R^2 = 0.457$			

Reference categories (Sex:F)

CI confidence interval

R^2 coefficient of determination

The regression coefficients were exponentiated.

Bold font denotes $p < 0.05$
