# Supplementary material for: Phenotypic Remodeling of γδ T Cells in Non-Eosinophilic Chronic Rhinosinusitis with Nasal Polyposis
Source: Medicina (Kaunas). 2025 Nov 30;61(12):2143. doi: 10.3390/medicina61122143 (PMC12734732; doi:10.3390/medicina61122143)
Supplement: Supplementary file 1 [file medicina-61-02143-s001.zip › Supplementary Figures_correction_28.11.2025.pdf]

Phenotypic Remodeling of γδ T Cells in Non-Eosinophilic Chronic Rhinosinusitis with Nasal Polyposis

Vjeran Bogović [1,2]†, Mario Štefanić [3]†, Stjepan Grga Milanković[1,2,] ,Željko Zubčić[1,2], Hrvoje Mihalj[1,2], Stana Tokić[4,*], Martina Mihalj[5,6,*]

Supplementary Figures.

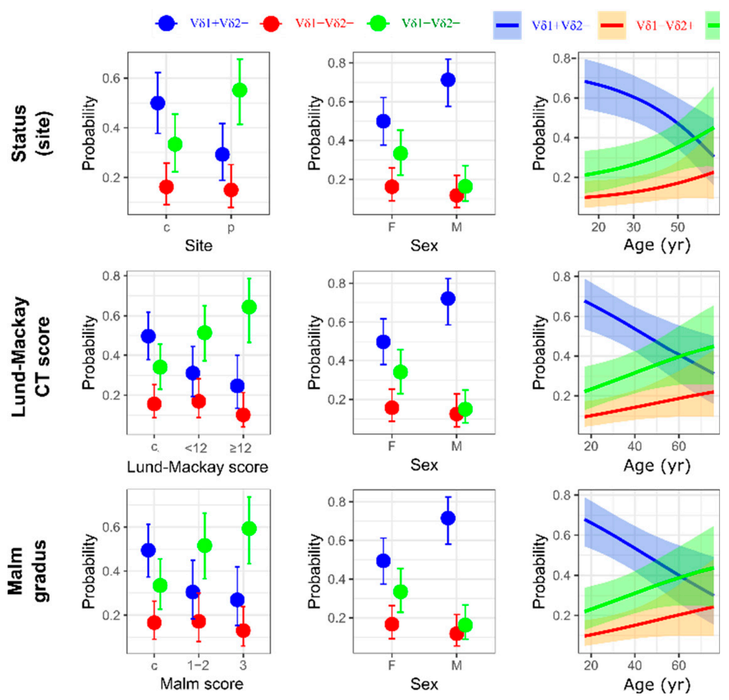

Supplementary Figure S1. Conditional (covariate-adjusted, independent) effects (mean, 95 % credible interval, posterior_predictions). Bayesian Dirichlet-multinomial regression was performed on compositional data (the proportions of the γδ T cell pool occupied by each cell subset). Each row corresponds to one multivariate model (fixed effects). Site denotes case (N=19) - control (c, N=10) status, polyps (p) *vs.* healthy mucosa. Color corresponds to cell type (horizontal labels, upper row). The shaded areas correspond to the 95% credible intervals. CT computed tomography. F females, M males. For average marginal predictions, see also Supplementary Table S5 (source data). Yr years.

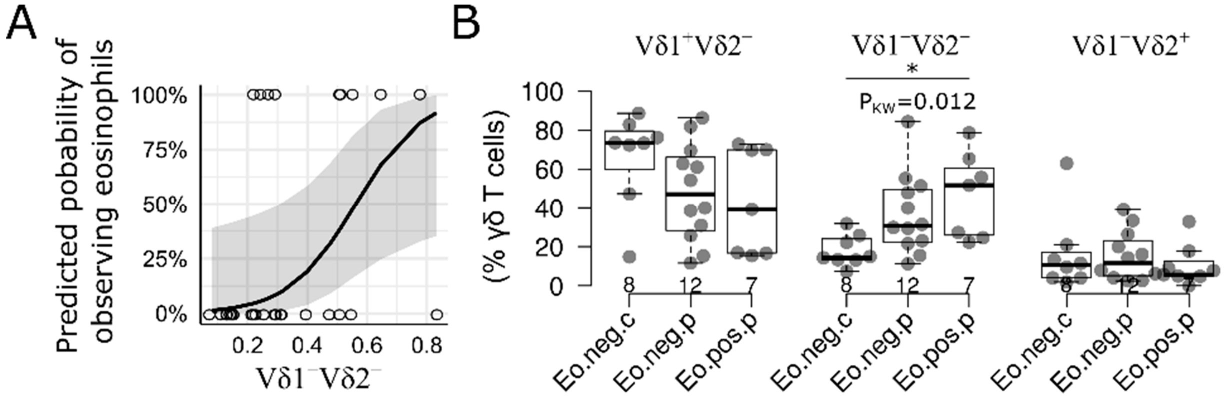

Supplementary Figure S2. A) The relationship between the proportion of $V\delta1^{-}V\delta2$ $\gamma\delta$ T cells in the nasal mucosa (parent = total $\gamma\delta$ T cells) and the probability of eosinophil shedding in nasal smears. (Y-axis, mean – solid curve, 95 % confidence interval – shaded area, binomial regression, $p<0.05$). Each circle corresponds to one donor (pooled sample of cases and controls, N=29). Details on regression models and their numerical solutions are available as source data (Supplementary Table S5 and Supplementary Table S8).

B) The raw data were split by case-control status according to the presence (pos.) or absence (neg.) of eosinophils (Eo) in nasal smears. A Kruskal-Wallis test was applied to each cell subset. Only significant results are indicated. An asterisk denotes *post-hoc* Conover $P<0.05$. c controls, p polyps. There were too few Eo.pos. controls (n = 2) for subgroup analysis. Boxplots are defined by medians and their respective interquartile ranges (IQRs). Vertical lines extend to ±1.5 IQR.

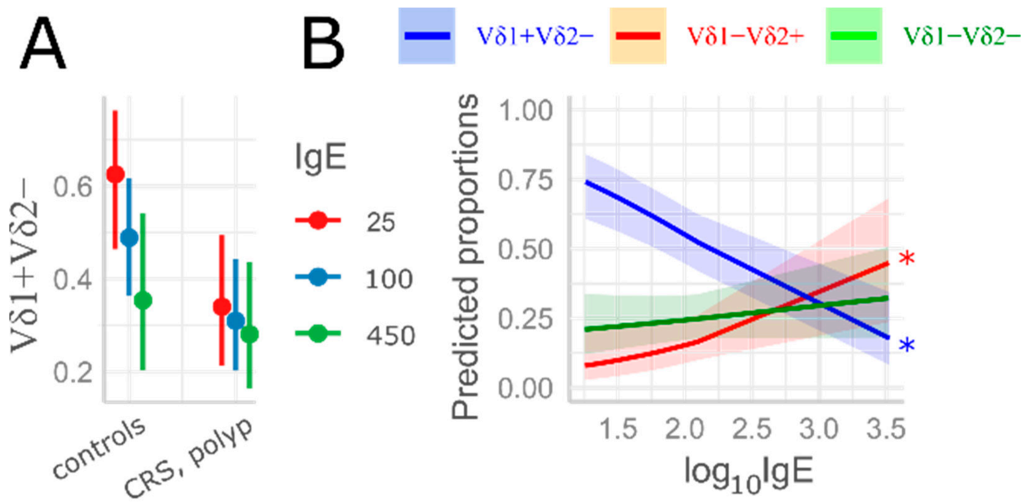

Supplementary Figure S3. Marginal means (95 % confidence interval), generalized linear model, beta regression. The relationship between log-transformed serum immunoglobulin E (IgE) levels (pg/mL) and the numerical abundance of the Vδ1⁺Vδ2⁻ γδ T cell fraction in the nasal mucosa (age and sex-adjusted model). The effect was split by case-control status (interaction term, panel (**A**), for three different IgE concentrations. Panel (**B**) shows the predicted proportions of γδ T cell subgroups in healthy donors. An asterisk (*) denotes a significant effect (p<0.05); color corresponds to cell type. CRS: chronic rhinosinusitis with nasal polyps. The shaded area corresponds to the 95% confidence interval. For details, see source data, Supplementary Table S9.
